# Consumer perspectives on simplified, layered consent for a low risk, but complex pragmatic trial

**DOI:** 10.1186/s13063-022-07023-z

**Published:** 2022-12-28

**Authors:** Tanya J. Symons, Nicola Straiton, Rosie Gagnon, Roberta Littleford, Anita J. Campbell, Asha C. Bowen, Adam G. Stewart, Steven Y. C. Tong, Joshua S. Davis

**Affiliations:** 1 Department of Medicine and Health Northern Clinical School, The University of Sydney, Sydney, Australia; 2 Faculty of Medicine and Health, The University of Sydney, Sydney, Australia; 3 Middlesex University, London, UK; 4 Centre for Clinical Research, Faculty of Medicine, University of Queensland, Royal Brisbane and Women’s Hospital Campus, Brisbane, QLD Australia; 5 Department of Infectious Diseases, Perth Children’s Hospital, Nedlands, Australia; 6 Wesfarmers Centre for Vaccines and Infectious Diseases, Telethon Kids Institute, Nedlands, Australia; 7 Division of Paediatrics, School of Medicine, University of Western Australia, Perth, Australia; 8 Victorian Infectious Diseases Service, The Royal Melbourne Hospital, at the Peter Doherty Institute for Infection and Immunity, Melbourne, Australia; 9 School of Medicine and Public Health, The University of Newcastle, Newcastle, Australia; 10 Infection Research Program, Hunter Medical Research Institute, Newcastle, Australia; 11 Menzies School of Health Research, Charles Darwin University, Darwin, Australia

**Keywords:** Informed consent, Consumer perspectives, Participant information sheet, Low-risk trials

## Abstract

**Background:**

For decades, the research community has called for participant information sheets/consent forms (PICFs) to be improved. Recommendations include simplifying content, reducing length, presenting information in layers and using multimedia. However, there are relatively few studies that have evaluated health consumers’ (patients/carers) perspectives on the type and organisation of information, and the level of detail to be included in a PICF to optimise an informed decision to enter a trial.

We aimed to elicit consumers’ views on a layered approach to consent that provides the key information for decision-making in a short PICF (layer 1) with additional optional information that is accessed separately (layer 2). We also elicited consumers’ views on the optimal content and layout of the layered consent materials for a large and complex Bayesian adaptive platform trial (the SNAP trial).

**Methods:**

We conducted a qualitative multicentre study (4 focus groups and 2 semi-structured interviews) involving adolescent and adult survivors of *Staphylococcus aureus* bloodstream infection (22) and their carers (2). Interview transcripts were examined using inductive thematic analysis.

**Results:**

Consumers supported a layered approach to consent. The primary theme that emerged was the value of agency; the ability to exert some control over the amount of information read before the consent form is signed. Three other themes emerged; the need to prioritise participants’ information needs; the importance of health literacy; the importance of information about a trial’s benefits (over its risks) for decision-making and the interplay between the two.

**Conclusions:**

Our findings suggest that consumers may challenge the one-size-fits-all approach currently applied to the development of PICFs in countries like Australia. Consumers supported a layered approach to consent that offers choice in the amount of information to be read before deciding whether to enter a trial. A 3-page PICF was considered sufficient for decision-making for the SNAP trial, provided that further information was available and accessible.

**Supplementary Information:**

The online version contains supplementary material available at 10.1186/s13063-022-07023-z.

## Background

Although informed consent is a key ethical requirement for research, questions remain about the utility of Participant Information and Consent Forms (PICFs) produced to support the trial consent process and document that consent has been received. PICFs should provide sufficient information to enable potential trial participants to understand the proposed research and the implications of participation. However, PICFs are often criticised for being lengthy and complex, for obscuring important detail, and for compromising understanding to an extent that calls into question the receipt of valid consent [1–3]. Clinical trial PICFs are also criticised for prioritising an institution’s need to mitigate risks over the needs of participants [4–6]. This is particularly evident when the risks and burdens of the trial are minimal, as is often the case for pragmatic comparative effectiveness trials comparing established interventions that would otherwise be available in clinical care. Conventional consent processes lead to two phenomena. The first, ‘injurious misconception’, where entry into a trial is rejected due to an exaggerated and disproportionate perception of risk [7, 8]. The second, ‘the nocebo effect’, where the way information is communicated, leads participants to experience side effects only because they expected them [9]. Consequently, researchers, bioethicists and consumers call for a more balanced representation of a study’s benefit-risk ratio [10, 11] especially for certain adaptive trials where there are increasing odds of benefit and reduced odds of harm as the trial progresses [12, 13].

Evidence suggests that a person’s understanding of the information in a PICF is inversely proportional to its length and that people prefer simpler forms [14]. However, despite the extensive literature describing interventions to address deficiencies in informed consent materials [5, 14–16], few data exist on the type and level of information people want to support their decisions to enter a trial [4, 5, 17].

The deficiencies in trial consent have also prompted more flexible and consumer-centric regulatory approaches. In the United States (US), for example, the revised Common Rule requires researchers to consider the information that ‘a reasonable person would want to have in order to make an informed decision about whether to participate’ (45 CFR 46.116 a [4]) and suggests this information is presented in layers, beginning with ‘a concise and focused presentation of the key information’ followed by more detailed information. However, even if information is sectioned in this way, prospective trial participants are often required to confirm they have read and understood the entire document before signing the consent form. Similarly, the Australian government has implemented the National Clinical Trials Governance Framework, [18] which extends the Partnering with Consumers Standard currently used for hospital accreditation, to an organisation’s clinical trial activity.

Guidance from the United Kingdom (UK) Health Research Authority (HRA) for pragmatic trials, extends the concept of ‘layered consent’ in one important way — trial participants can sign the consent after reading only the *first layer* (a concise PICF), if this PICF links to supplementary information in a *second layer*, (e.g., a trial website or separate written document) for those who want to read more. The HRA guidance also recognises that for ‘point of care’ pragmatic trials, the clinical consent discussion would serve to supplement the written information for the trial. The RECOVERY trial (NCT04381936), a high-profile COVID-19 trial of repurposed treatments that has recruited over 48,000 international participants, has used many of the principles of layered consent, embracing reduced length (3 pages + consent form), and links to further optional information on the trial’s website, including data protection regulations. By contrast, a one-size-fits-all approach to PICF development is widely adopted in Australia, where standardised national templates published by the National Health and Medical Research Council (NHMRC) are often used [19]. Researchers seldom depart from the ‘preferred’ wording provided in this template, possibly for fear that it will delay study approval.

To operationalise the HRA’s concept of layered consent, the PICF must contain sufficient information for valid consent [20] and comply with local regulatory requirements. Therefore, our study objective was to elicit consumer views on layered consent and the content suitable for each layer. We tested the concept with the draft PICF developed for the SNAP trial, a complex, adaptive platform trial comparing routine treatments for *Staphylococcus aureus* bloodstream (*SAB*) infections. We elicited the view of consumers with lived experience of the disease condition under study.

## Methods

This was a qualitative, descriptive exploratory study using focus groups and interviews to elicit consumer views on layered consent. Four 2-h focus groups were held, one in each participating site. Three were held prior to COVID-19 as face-to-face sessions at each organisation. The study was amended in 2020 to enable the last focus group to be held online and two telephone interviews to be conducted in early 2021. Adults and adolescents historically hospitalised for bloodstream infections (along with their carers) were recruited over a 16-month period.

Two female members of the research team, TS for the adult focus groups and interviews and AC (for the paediatric and parent focus group), both experienced facilitators, led the sessions. TS is a researcher and trial consultant who is external to the SNAP trial. AC is an infectious disease physician not involved in the direct care of any of the study participants. A semi-structured interview guide (see Additional file 1) was developed and reviewed by members of the research team and a consumer representative. An investigator from the SNAP trial attended each focus group to answer questions about the trial.

The 3-page PICF for the SNAP trial (see Additional file 2) was developed with input from three consumer representatives, one with a background in writing educational materials, one with experience working in health, and a prior SAB survivor. The PICF contained the basic elements of consent required by Sect. 2.2.2 of the National Statement on Ethical Conduct in Human Research [21] which states:

Participation that is voluntary and based on sufficient information requires an adequate understanding of the purpose, methods, demands, risks, and potential benefits of the research.

This aligns with both the expert analysis of the disclosure requirements for consent in pragmatic trials [22] and the key information elements identified in the preamble to the US Common Rule [23].

### Study participants

All consumers that participated in this study were a purposive sample of SAB survivors (or carers of SAB survivors) recruited from three cohorts — those who participated in a previous randomised controlled trial on SAB at the study sites [24], those who had been treated in hospital or via the local Hospital in the Home program for > 1 week and those from an observational cohort of children and adolescent patients with SAB [25]. Consumers were English-speaking adults and adolescents.

Participants were contacted by letter (between 25 and 50 letters per site were dispatched) with a phone number and reply slip so that they could opt out from any further contact if desired. After 2 weeks, individuals who did not opt out were contacted with an explanation of the main study goals and discussion points.

One hundred and forty invitations were sent from which thirty-four consumers agreed to participate and were sent pre-reading material, including a copy of the SNAP PICF. Twenty-four consumers attended the sessions: six in Group 1, twelve in Group 2, three in Group 3, one in Group 4 and two by telephone. No further information or feedback was collected from those who participated or from non-attendees. No repeat interviews were carried out. Demographics for attendees are outlined in Table 1.

**Table 1:** Characteristics of focus group/interview participants

**Characteristic**	
**Sex**	
Male	18 (75.0)
Female	6 (25.0)
**Age (years)**	
Range	10–85
Median (IQR)	54.5 (41.5–65.3)
**Ethnicity**	
Caucasian	12 (50.0)
Aboriginal/Torres Strait Islanders	1 (4.2)
Asian	2 (8.3)
Unknown	9 (37.5)
**Education: adult attendees** [22]	
University degree	6 (25.0)
No university degree	10 (41.7)
Not known	6 (25.0)

### Focus groups and interviews

Each focus group began with an education session, which included an extract from a short video developed by the UK Health Research Authority as part of a public dialogue project on recruiting people into health research: Conversation Simplified Consent [26]. The video describes randomised controlled trials, the concept of a pragmatic trial involving routinely used treatments, the current research consent process, and the rationale for embedding trials into routine care. The consumers interviewed by telephone had this information described to them. Consumers then received information about the SNAP trial and the concept of layered consent was described. The PICF was read aloud to consumers, who were informed that it was the first layer of information for the SNAP trial, and that the trial’s website would contain a second layer of optional information. Consumers then provided views on layered consent and the PICF, which included their views on the inclusion of a benefits statement applicable to adaptive trials. Two alternative statements were included (see Additional file 2), and consumers were asked whether either of these statements was relevant to their decision-making, and which statement was preferred. The primary objectives were to gather consumer views on the following:

1. The acceptability of layered consent
2. Whether the SNAP PICF contained sufficient information for decision-making and whether there were any elements that should be added to/removed from the PICF
3. Whether inclusion of a ‘benefit statement’ in the PICF applicable to platform trials, was relevant to a person’s risk–benefit assessment.

### Analysis

The focus groups and interviews were audio recorded, and the recordings were transcribed verbatim and verified for accuracy. The transcripts were analysed. Codes and themes were identified using inductive thematic analysis [27] and recorded using NVIVO v12 by NS. NS and TS analysed all transcripts, compared coding schemes, reviewed discrepancies, and discussed and agreed on all themes and the point at which data saturation had been reached.

## Results

Layered consent was strongly supported, and consumers considered the 3-page PICF was sufficient to make an informed decision. The major theme that emerged was the sense of agency the layered approach provided; the ability to choose content that was most relevant to them and to sign the consent without reading the second layer if they considered the information in the PICF sufficient. Consumers felt that keeping the PICF short had a direct bearing on the likelihood they would read and understand the document. In addition, three other themes emerged: (1) the need to prioritise participants information needs in trial policy, (2) the relevance of knowledge about the health condition under study to support the decisions about trial participation, and (3) the importance of providing information on a trial’s benefits over risks, including consideration for the wording in these sections so as not to overestimate risks. Feedback on layered consent and the PICF for the SNAP trial and the themes that have emerged are described below with illustrative quotes.

### Support for layered consent and the value of agency

Overall, the majority of consumers supported the use of layered consent for the SNAP trial, primarily because they felt it provided a greater sense of agency:

I like the idea of just a little bit of information with the option of getting more

IN all sessions, the desire to choose the amount of information read was expressed in terms of the differing information needs of patients.

…the difference is between people who need a lot of information…and people who are quick to say that all makes sense, it’s [layered consent] in the general interest of everybody.

Several consumers reflected that layered consent can be particularly helpful in periods of acute illness when the ability to receive and process information may be sufficient for valid consent, but nonetheless impaired.

…to have something simple, basic, it’s the best way to go, especially when you’re sick. The last thing you want to do is to read an encyclopaedia.

Support for a layered approach to consent that promotes choice was also articulated in two focus groups from the perspective of a carer:

I’m just looking at it from a family perspective. When my brother was in hospital, he was not really well enough to make the best decisions. So having the short one [PICF] would be right for him in that mental state…and to have an information pack that you could give to the family, that would be important.

One parent commented that their immediate response, if approached for a trial in a time-critical environment, would be negative due to the stress of the situation, but that a concise first layer could overcome this barrier:

…if you can break the barrier or if you can quickly move past that then I get the feeling that this [PICF] might be, you know, might be it rather than 10 or 20 pages

Despite the majority of consumers in this study supporting layered consent, potential concerns were raised. For example, some cautioned that the second layer of information should be easily accessible and presented in a way that allows the option to select only the content that interests them:

…I think your layered system gives you that [options] right? Provided you can pick the bits of the layer that you want. It [the website] might need to have headings.

### Prioritising participants’ needs

In four sessions, consumers expressed an awareness that there may be legal reasons why their choice or agency may be restricted, but another theme that emerged was the mismatch between what is required, and what potential trial participants might want or need. Consumers felt that whilst there are regulations that govern trials, the values and preferences of participants (and their carers) should be of primary consideration:

The ethics committee will look at legal liability… but at the same token, they've got to look at our side of it and not their side of it.

…32 pages that probably make the lawyers feel sensational… in my view, apart from the legality of it, there’s little to no value at all…because nobody will read it

The majority of consumers confirmed for them, the PICF (layer 1) provided all the information they needed to make an informed decision about entry into the trial:

I don’t like to read much. But I think that holds all the information that I'd need to make a decision.

The need to ensure information in the PICF is presented clearly was raised. There was strong support for the bulleted format, but less enthusiasm for diagrams:

…it’s all in point form so it’s clear and precise. I mean, point form’s the key, short, sweet point form.

…if you have bullet points, it’s much better because the ones that I have seen, they just, um, it’s just continual sentences saying, you know, this this and this, and you’ve really got to read into it before you can understand it properly… Diagrams just confuse people, whereas bullet points are more in your face.

### Knowledge of the condition under study

Several consumers reported that a lack of understanding of their health condition due to inadequate information during clinical care would affect their decision about trial participation. Although this was recognised as a failure in communication during clinical care rather than research, consumers suggested that research consent would present another useful opportunity to improve knowledge about a potential trial participant’s disease condition:

I think I would want to know a bit more about my physical health and the long-term things about the antibiotic…

I thought on the video, where it was explaining that it lives on the skin that could be included [in the PICF]

### Research benefit before risk

Consumers were asked their views on the addition of a benefits statement applicable to adaptive trials. Two alternatives were included in the PICF. They were also asked whether such statements would be relevant to their decision-making, and which was preferred.

The majority preferred the following statement, with several confirmed it was relevant to their risk–benefit assessment and overall decision-making:

This study is called an Adaptive Platform Trial. In this type of study, the researchers analyse the results as the study goes on rather than just at the end. This means that people who take part in the study once it has been running for a while have a better chance of getting a better treatment.

When risks and benefits were discussed, a final theme that emerged was the importance of the clear articulation of a trial’s benefits over its risks:

They want to know why they’re doing this in the first part. You know, I’d actually have it [the benefits section] higher up the list… I'd have it as number 2. Because the chances of people reading the first three are higher…

Several consumers suggested the word ‘trial’ should be avoided when routine care interventions are tested, so as not to overinflate risk, recognising that risks are ‘kept to a minimum’ in this type of trial:

… make it clearer that you are not asking them to try a new medicine, you are asking them to record the effects of the medicine they are going to get anyway, essentially.

…the thing that still worries me a little bit, is the word ‘trial’ - is there any other way that I can get rid of that word? … these antibiotics are tried and tested, I mean, to me, if I’m lying-in bed crook as a dog and somebody comes in and says, we’re going to trial something, first thing I think of is, well I'm I the local guinea pig.

## Discussion

We found strong support for layered consent. Firstly, consumers valued the sense of agency it provided, allowing control over the amount and type of information read during the consent process and thus, accounting for the wide variation in their information needs. Secondly, it supported the creation of more concise PICFs, which they believed were much more likely to be read and understood.

Consumers recognised that organisations (through their legal processes and governance policies) need to manage risk, but as illustrated in their quotes, consumers were critical that this took precedence over their values and preferences. In Australia, the National Statement requires trials to be grounded in four ethical principles—research merit and integrity, beneficence, justice and respect, and that researchers have ‘due regard for the welfare, beliefs, perceptions, customs and cultural heritage…of those involved in research’. Consumer preferences regarding PICF content are therefore paramount, but have rarely been included in this level of discussion. But although the SNAP PICF incorporates consumer views and preferences, it may still draw criticism, as it does not include content specified in Sect. 4.8.10 of the Good Clinical Practice (GCP) Guidelines [28]. However, GCP was specifically developed for trials submitting data to regulatory authorities for licensing of new or investigational drugs, and the Guideline itself states that compliance with its principles is more appropriate for other trial types. Moreover, full compliance is not a legal requirement in many regions, including Australia [29]. The SNAP trial (NCT05137119) is now approved (HREC/74098/MH-2021) and recruiting patients in Australia, New Zealand and Canada using a final PICF based on the PICF co-designed by our consumers (see Additional file 3), suggesting that ethics committees are also comfortable with layered consent.

Consumers supported a bulleted format for the PICF, consistent with studies that have elicited consumer and expert feedback [3, 10, 30]. Many felt that bullet points made the PICF easier to read and to locate the information most important to them. There was, however, less support for the diagrams, although this finding may have been due to the complexity of the information provided in the diagrams that were included.

Our findings highlight the importance of adequate knowledge of a patient’s disease condition when decisions about trial participation are made, suggesting that poor understanding of the health condition may hamper the ability to make well-informed decisions about trial participation, especially in acute conditions. This finding aligns with prior research suggesting that patient awareness of the health problem being studied is a precursor to successful trial recruitment [14, 31]. In large trials like SNAP with a dedicated website, there are opportunities to enhance understanding using multimedia learning resources [32]. Finally, consumers supported the inclusion of a ‘benefits’ statement in the PICF relevant to platform trials, as it was considered important for decision-making. They also supported a more balanced description of the trial’s risk–benefit ratio, so that risks are not overinflated.

The information provided to prospective participants for individual trials is context dependent. It is possible, therefore, that different conclusions would have arisen and in study populations with lower acuity medical conditions or in other settings (our consumers were drawing on their experience of being hospitalised or their experience having their children or relatives hospitalised). However, this study provides insights into the information a person needs when considering trial enrolment and in Australia in particular, the findings are likely to be applicable, as the key topics for discussion in the NHMRC PICF templates were included.

The study also reinforces the value of consumer involvement in the development of PICFs, not only to improve the information contained in the PICF, but also to reassure ethics committees that consumers consider layered consent to be a suitable approach. Moreover, many commentators in the US have concluded the *reasonable person* standard in the Common Rule should be operationalised for each trial with the help of consumers [23, 33].

A strength of this study is that it tested layered consent with consumers with experiential knowledge of the disease condition under study. Despite the absence of field notes, the use of focus groups and interviews was an effective way of drawing rich insights on complex issues like optimising trial consent, both from the perspective of patients and carers. We recognise, however, that the view of patients or carers considering trial participation and differ from our focus group participants. Our study has several other limitations. Only 24 consumers took part, in part because recruitment in the final focus group, which took place early in the pandemic, proved challenging. However, additional focus groups were discounted as our inductive thematic analysis indicated saturation had been reached. We also acknowledge that despite efforts to avoid framing effects when eliciting people’s responses, it is impossible to discount that their responses were impacted by the conversational frame. Finally, although efforts were made to ensure our cohort was diverse, few were from different cultural and ethnic populations and the sample was dominated by male participants and therefore did not fully represent the diversity of the Australian population.

## Conclusion

Health consumers with lived experience of Staphylococcus aureus blood stream infection support layered consent; a novel approach where reading some of the ‘fine print’ normally contained in PICFs is optional, allowing consent to be signed after reading only the first layer. Their views informed the layered consent approach used in the SNAP trial.

Key themes from this study included the value of agency, the need to incorporate consumer’s values and preferences into trial policy, the relevance of knowledge about the health condition under study to support decisions about trial participation, and the need to avoid overinflating risk in low-risk trials.

Consumer-centricity is now a central requirement of government-funded trials. Ethical guidelines also require trial consent to be grounded in the principle of respect, which requires consideration of consumer views. We suggest that the co-design of consent materials with consumers improves their utility and ethical defensibility and should be considered in the planning of clinical trials, particularly those of complex design.

## Supplementary Information

**Additional file 1. **Semi-Structured Moderator’s Guide. Used for the focus groups and interviews conducted in the study.

**Additional file 2. **Draft Participant Information Sheet. consent form removed.

**Additional file 3. **Final Participant Information Sheet Approved by the Ethics Committee Reviewing the SNAP Trial. consent form removed.

## Data Availability

Anonymised transcripts are available from the corresponding author on request.

## Abbreviations

PICF: Participant information sheets/consent form
SNAP: *Staphylococcus aureus* Bacteraemia Network Adaptive Platform Trial
US: United States
UK: United Kingdom
HRA: Health Research Authority
NHMRC: National Health and Medical Research Council
SAB: *Staphylococcus aureus* Bloodstream
