# Supplementary material for: Consumer perspectives on simplified, layered consent for a low risk, but complex pragmatic trial
Source: Trials. 2022 Dec 28;23:1055. doi: 10.1186/s13063-022-07023-z (PMC9795139; doi:10.1186/s13063-022-07023-z)
Supplement: Supplementary file 1 — Additional file 1. Semi-Structured Moderator’s Guide. Used for the focus groups and interviews conducted in the study. [file 13063_2022_7023_MOESM1_ESM.pdf]

ADDITIONAL FILE 1

Semi-Structured Moderator's Guide

Used for the focus groups and interviews conducted in the study

Moderator's Guide

WELCOME AND GROUND RULES

We are conducting several focus groups to hear people's thoughts on simplified consent to enter a clinical trial called SNAP which is looking at treatments for Staph aureus bacteraemia (SAB)

- *We have invited you to participate in this focus group because you a familiar with what it is like to have a SAB infection and therefore can give us unique insights into some of the questions the researchers have about a study involving SAB patients*
- *We will use the information we gather to help us produce the participant information leaflet that will be used for SNAP*
- *My role is to guide the discussion today.*
- *There are no right or wrong answers, only opinions*
- *Please feel free to share your ideas and opinions, even if they differ from what others have said. It's helpful to hear different points of view*
- *Everything you say will be confidential - we won't use any names or other information that could identify you in our reports. A small number of named researchers will have access to the recordings.*
- *We'd like to hear from all of you equally*
- *Also, feel free to talk to each other as well as to me. If at any time during our discussion you don't feel comfortable answering a question you don't have to*
- *Please be respectful of others' privacy*
- *Because we have limited time together, I may, for the sake of time, jump in and move the discussion forward*
- *If you have any questions about our research or how your feedback will be used, please feel free to speak with me after the session.*
- *We will stop for a short break at [time] but feel free also to get up and get more refreshments and to use the bathroom*
- *Please turn off any mobile phones or other electronic devices until we are finished*
- *As we mentioned when we contacted you, we are audio recording the meeting so that your comments are accurately captured during our discussion.*

[Confirm verbal agreement to proceed] *Thank you.*

PARTICIPANT WARM-UP

ICE BREAKER

EDUCATIONAL COMPONENT AND QUESTIONS

Before getting into our main discussion, we would like to give you a little more background information

Introduce researcher to presents information about the SNAP trial (2-3 slides)

Short video clip on simplified consent

Present content (role of ethics committee and concept of layered consent as a possible way to simplify consent).

QUESTION: What are your first thoughts on layered consent?

PROMPTS (e.g. ask if any concerns if none raised initially)

QUESTION: Do you think ethics committees should encourage researchers to use layered consent?

PARTICIPANT INFORMATION SHEET

Read Information Sheet (and project on screen)

CONTEXT: Do you consider yourself the kind of person who is not very interested in detail, or a person who likes to read all available information before making a decision? Let's go around the room and check.

COMFORT BREAK

The information sheet we have read to you represents the first layer of information for someone considering taking part in a trial. This means it should contain just enough information to make an informed decision whether or not to take part.

Explain how more information can be accessed for those who need it i.e. verbally from research team and from trial website which contains all the additional information generally required in a traditional PICF.

QUESTION: Do you think this information sheet contains enough information to make a decision to go into the trial?

PROMPT: Is there anything that could be taken out because it is not needed to make a decision. Is there anything that is missing that needs to be in the document for a patient to make a decision?

PROMPT: As necessary

QUESTION: What are your thoughts on the layout or presentation of the information leaflet?

PROMPT: As necessary.

I would like to ask your thoughts on presenting information on 'trial benefit'.

Traditional trials usually compare, say, treatment A with treatment B, and then another traditional trial compares treatment B with treatment C. A platform trial compares lots of different treatments at the same time. Unlike traditional trials, researchers analyse the information collected as the trial goes on. The treatment that are not performing well are dropped. Those treatments would still be being used outside the trial in routine care until well after the study has finished.

What this means is that the chance of getting a treatment that is not so effective reduces as the trial progresses.

QUESTION: For platform trials, should the information sheet include a statement like one of these:

“For patients not taking part in this study, the choice of antibiotic is made according to the preference of the local medical team. This approach to care may involve more risk than being in a study like SNAP which involves a carefully designed protocol and close monitoring to ensure that as the trial progresses, there is a lower chance of receiving less effective treatments.

‘This study is called a Platform Trial. In this type of study, the researchers analyse the results as the study goes on rather than just at the end. This means that people who take part in the study once it has been running for a while have a better chance of getting a better treatment.’

QUESTION: [If yes] Which one do you prefer?

PROMPT: Why?

WRAP UP

Thank attendees and give out gift voucher.

Explain that we would like to be able to send the final PICF produced after all focus groups have been completed so that 1) they can see their contribution and 2) they can confirm that in their opinion, it contains sufficient information to make a decision to participate in a trial (asked to contact the site if not).
