# Supplementary material for: Consumer perspectives on simplified, layered consent for a low risk, but complex pragmatic trial
Source: Trials. 2022 Dec 28;23:1055. doi: 10.1186/s13063-022-07023-z (PMC9795139; doi:10.1186/s13063-022-07023-z)
Supplement: Supplementary file 2 — Additional file 2. Draft Participant Information Sheet. consent form removed. [file 13063_2022_7023_MOESM2_ESM.pdf]

ADDITIONAL FILE 2

Draft Participant Information Sheet Tested with Consumers

(Consent form removed)

An International Study of Commonly Used Antibiotics to Treat 'Golden Staph' Infections

(The *Staphylococcus aureus* Network Adaptive Platform (SNAP) Trial)

INFORMATION FOR PARTICIPANTS

We are inviting you to take part in a research study. Please read this information which will help you decide.

You and your doctor have agreed that you would benefit from antibiotic treatment for your "golden Staph" blood stream infection. Doctors call this type of infection Staphylococcus Aureus Bacteraemia (or SAB for short), There are three types bacterium (germ) that cause SAB.

- Penicillin-susceptible *S. aureus* **(PSSA)**
- Methicillin-susceptible *S. aureus* **(MSSA)**
- Methicillin-resistant *S. aureus* **(MRSA)**

Your SAB infection is being caused by one of these germs

1. Why are we doing this study?

- Many kinds of antibiotic are used to treat SAB. All of these antibiotics are safe and work well.
- This study aims to find out which antibiotic (or combination of antibiotics) work best for each type of SAB infection.

2. Do I have to take part?

- If you do not want to take part that's OK. Your decision will not affect the quality of care you receive.
- If you decide *not* to take part, you will be given the standard antibiotic treatments used in this hospital. This may be the same treatments you would have received in this study.
    - If you take part, you are free to withdraw at any time without giving a reason.

3. If I take part, what will I need to do?

- You will be asked to sign a consent form.
- You do not need to do anything more. There are no extra visits or tests.
- We will collect all the information needed (like blood results) from your medical records.
- You will be followed-up for 90 days from the time you start treatment. The whole study will last for 5 years.

4. What will the study involve?

- You will also be randomly allocated (like flipping a coin) to your main treatment(s) which will depend on the type of SAB infection you have. This part of the study will help us find out which of the currently used antibiotics works best.

- You will also be randomly allocated to have an antibiotic called Clindamycin added to your main treatments, or not. Doctors do not know whether adding Clindamycin will make people recover more quickly. This part of the study will help us find out.

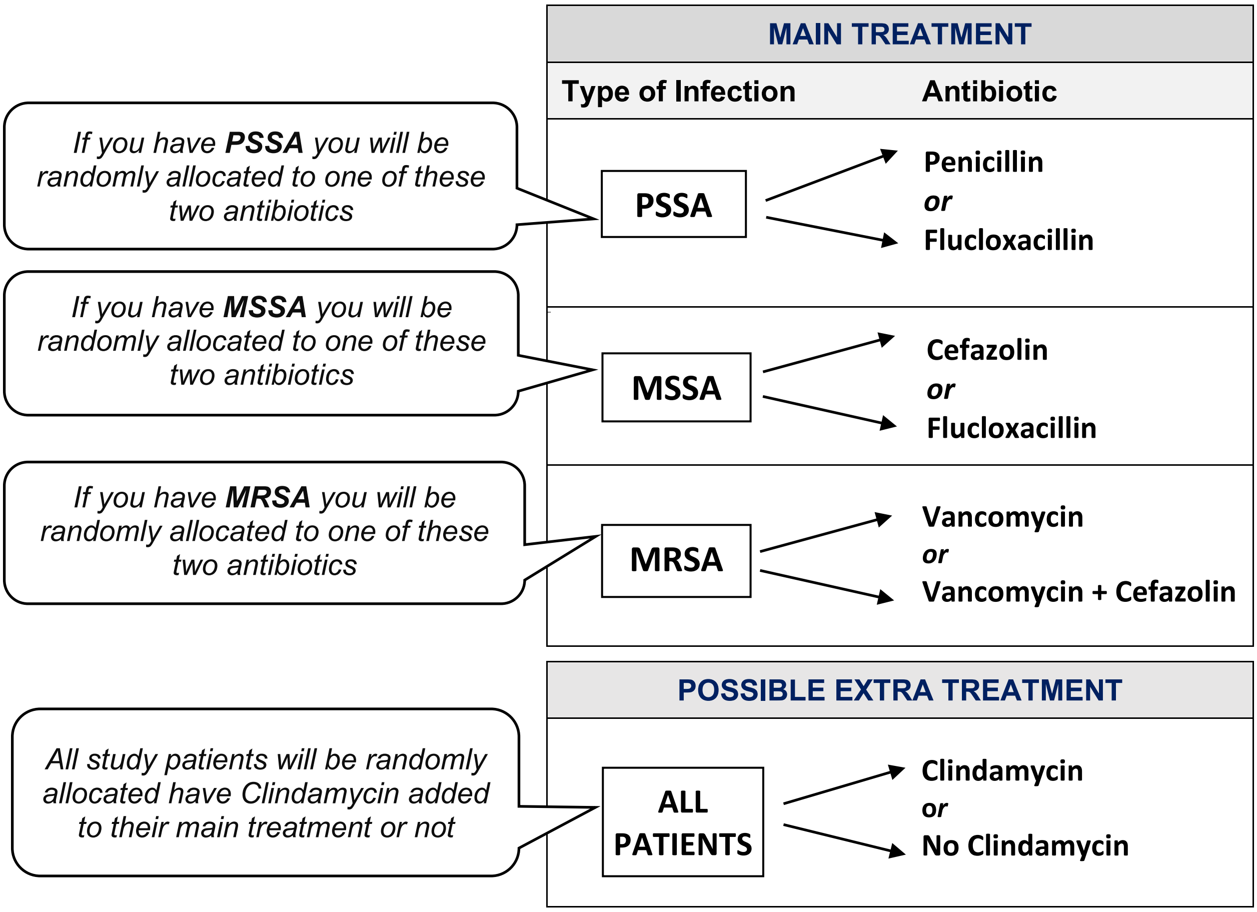

5. What are the risks of taking part?

- All the treatments you may receive in this study are already widely used in normal care. The risks of being in the study are low.
- All antibiotics may have side effects. Most side effects are mild, such as diarrhoea, thrush and rash.
- Very rarely, more severe side effects might happen, such as liver or kidney impairment or muscle damage, but these usually get better when the treatment is stopped.
- Your doctor will rule out any antibiotics you should *not* take based on your medical history and also, for women, whether you are pregnant

6. What are the benefits of taking part?

- You may not benefit from this study, but it is possible that the treatment you receive may work better, or more quickly than the treatment that you would have received if you did not take part.
- For patients not taking part in this study, the choice of antibiotic is made according to the preference of the local medical team. This approach to care may involve more risk than being

- in a study like this, which involves a carefully designed protocol and close monitoring to ensure that as the trial progresses, there is a lower chance of receiving less effective treatments.
- This study is called a Platform Trial. In this type of study, the researchers analyse the results as the study goes on rather than just at the end. This means that people who take part in the study once it has been running for a while have a better chance of getting a better treatment.
- Research like this also helps to continually improve the treatments and care provided to all patients now and in the future.

7. What will happen to the information collected for this study?

- Your information will be kept strictly confidential. The only people allowed to look at information that could identify you (such as your name and address) will be your doctors, study staff and regulatory authorities who may want to check that the study is being carried out correctly.
- Your doctor will use this to contact you for the 90-day follow up check, and to send you information about the study results.
- We may link your details to existing data (e.g., Medicare) to allow longer follow-up.

8. What will happen to the samples collected for this study?

- The bacteria grown from your blood culture will be stored in a sample bank.
- It will be used to help researchers better understand the factors that make antibiotics work well on certain types of people and not others.

9. Who is organising this study?

- The study is being led and organised by …………
- The study is being funded by a research grant from the National Health and Medical Research Council. The lead organisation is ……

10. Where can I find more information?

- Your doctor can answer any questions you may have.
- You can also go to trial's website [ADD] to find out more information including:
  - More information on each of the antibiotics in the trial
  - More on how your privacy will be protected
  - Details of the ethics committee that approved the study
  - Compensation arrangements in the unlikely event that anything goes wrong.
  - More information about clinical trials in general.

Thank you for taking time to read this information sheet

How to contact us
Here is the contact for your study doctor:<br><br>Dr X or Dr Y<br>Telephone:
